# Activation of α7-nAChRs Promotes the Clearance of α-Synuclein and Protects Against Apoptotic Cell Death Induced by Exogenous α-Synuclein Fibrils

**DOI:** 10.3389/fcell.2021.637319

**Published:** 2021-02-25

**Authors:** Jifeng Zhao, Yun Li, Yan Li, Shi Xu, Tingting Tao, Ye Hua, Ji Zhang, Yi Fan

**Affiliations:** ^1^Department of Pharmacology, Neuroprotective Drug Discovery Center of Nanjing Medical University, Nanjing Medical University, Nanjing, China; ^2^Department of Neurology, Affiliated Wuxi Clinical College of Nantong University, Wuxi, China; ^3^Division of Clinical Pharmacy, Department of Pharmacy, The First Affiliated Hospital of Nanjing Medical University, Nanjing, China

**Keywords:** α-synuclein, apoptosis, Parkinson's disease, PNU-282987, nicotinic acetylcholine receptor

## Abstract

Misfolding and abnormal aggregation of α-synuclein (αSyn) have been shown to increase the risk of developing Parkinson's disease (PD). Finding some way to reduce the aggregation of αSyn is particularly important for the treatment of PD. The main route in prion-like αSyn spreading is the cholinergic innervated vagus nervous system and central cholinergic neurons. Since the degenerative changes and death of cholinergic neurons also run through the pathological process of PD, we hypothesize an involvement of the cholinergic system in αSyn aggregation. The α7 nicotinic acetylcholine receptors (α7-nAChRs) are one of the most abundant nAChRs in the mammalian brain. Using nicotine and a selective α7-nAChRs agonist PNU-282987, we found a protective effect of α7-nAChRs on the cell damage induced by αSyn-PFF (preformed fibrils) through inhibiting apoptotic cell death. We further discovered an additive effect of α7-nAChRs on the clearance of αSyn in normal and αSyn stably transduced SH-SY5Y cells. Moreover, using α7-nAChRs knockout mice, we noticed that α7-nAChRs deficiency increased the deposition of αSyn and aggravated the loss of dopaminergic neurons in a chronic MPTP mouse model of PD. Our findings for the first time indicated that α7-nAChRs activation exhibited a neuroprotective effect on αSyn pathology and aggregation by promoting the clearance of αSyn.

## Introduction

Parkinson's disease (PD) is the second most common neurodegenerative disease after Alzheimer's Disease (Nussbaum and Ellis, 2003). This disease is more common in the elderly, and the prevalence increases with age. As previously reported (Raza et al., 2019), about 1–3% of the population over the age of 65, and its number is going to rise from 8.7 to 9.3 million by 2030. The typical clinical manifestations of PD are motor symptoms (resting tremor, slow movement, rigidity, difficulty walking, and gait) and non-motor symptoms (autonomic disorders, smell, vision, sleep, cognition, and affective disorders) (Meissner et al., 2011; Rascol et al., 2011; Schapira and Jenner, 2011). The pathological characteristics of PD are the progressive loss of dopamine neurons in the substantia nigra and the appearance of Lewy bodies (LBs) and Lewy neurites (LNs) in neurons (Dickson, 2018). LBs and LNs are intraneuronal inclusions composed of α-synuclein (αSyn) aggregates. The presence of αSyn in cytoplasmic inclusions represents aberrant cytologic localization since αSyn is a natively unfolded protein enriched in presynaptic terminals and plays a role in synaptic vesicle release (Burré et al., 2010). Numerous studies have shown that αSyn misfolding and abnormal aggregation can cause severe damage to neurons and other cells, leading to mitochondrial damage (Devi et al., 2008; Luth et al., 2014), endoplasmic reticulum stress (Colla et al., 2012), synaptic dysfunction (Huang et al., 2019), autophagy, and lysosomal dysfunction (Nguyen et al., 2019). While misfolding and abnormal aggregation of αSyn is the pathological mechanisms of PD, finding some way to reduce the aggregation of αSyn is particularly important for the treatment of PD.

Many studies recently indicate that αSyn is transported in the long-distance and bi-directional gut-brain axis (Kim et al., 2019). This main transport route is the cholinergic innervated vagus nervous system and central cholinergic neurons (Travagli et al., 2020). In the distal gut, αSyn is selectively expressed in most of the intestinal cholinergic axons. Moreover, in addition to the progressive death of dopaminergic neurons, the degenerative changes and death of cholinergic neurons also run through the pathological process of PD (Perez-Lloret et al., 2016). These findings suggest an involvement of the cholinergic system in αSyn aggregation and transmission.

In the central nervous systems, cholinergic neurons and nicotinic acetylcholine receptors (nAChRs) participate in diverse functions. A large number of epidemiological studies showed a lower incidence of PD in tobacco smokers (Hernán et al., 2002; Thacker et al., 2007; Ascherio and Schwarzschild, 2016). Nicotine, a key ingredient in tobacco products, has neuroprotective effects of dopaminergic neuronal loss in PD models via acting on nAChRs (Quik et al., 2014; Perez, 2015). Among these nAChRs, α7-nAChRs are one of the most abundant nAChRs (Quik et al., 2015). Our previous studies found that α7-nAChRs activation has a protective effect on cell damage caused by 1-methyl-4-phenylpyridinium (MPP^+^) (Xu et al., 2019). However, the potential roles of α7-nAChRs in the cellular toxicity of αSyn aggregates have remained unknown.

In the present study, we prepared αSyn-PFF (preformed fibrils) formed from purified recombinant human wild-type αSyn and constructed SH-SY5Y cells overexpressing human wild-type αSyn. Since SH-SY5Y cells express endogenous α7-nAChRs subtypes (Peng et al., 1997), we used a non-selective nAChR agonist nicotine and a selective α7-nAChR agonist PNU-282987 to test whether their addition to SH-SY5Y cells challenged with αSyn-PFF could afford neuroprotection. We found that α7-nAChRs activation protected against apoptosis induced by αSyn-PFF and reduced the levels of αSyn in normal SH-SY5Y cells and stably transduced cells. Furthermore, using α7-nAChRs knockout (KO) mice, we discovered that α7-nAChRs deficiency increased the deposition of αSyn and aggravated the loss of dopaminergic neurons in a chronic regimen of 1-methyl-4-phenyl-1,2,3,6-tetrahydropyridine (MPTP) and probenecid (MPTP/p). Our findings for the first time indicate that α7-nAChRs activation can reduce α-synucleinopathies by promoting the clearance of αSyn.

## Materials and Methods

### Reagents

The ToxinEraser™ Endotoxin Removal Kit (#L00338) and ToxinSensor™ Chromogenic LAL Endotoxin Assay Kit (#L00350C) were purchased from GenScript (Piscataway, NJ, USA). MPTP, MPP^+^, nicotine, PNU-282987, methyllycaconitine citrate (MLA), puromycin, probenecid, 3-[4,5-dimethylthiazol-2-yl]-2,5 diphenyl tetrazolium bromide (MTT), JC-1, and TH (Tyrosine hydroxylase) antibody (#T1299) were purchased from Sigma-Aldrich (St. Louis, MO, USA). The cOmplete^TM^ EDTA-free protease inhibitor cocktail and PhosSTOP phosphatase inhibitor cocktail were purchased from Roche Applied Science (Indianapolis, IN, USA). LDH detection kit was obtained from Nanjing Jiancheng Bioengineering Institute (Nanjing, China). Hoechst 33342 was purchased from AAT Bioquest (Sunnyvale, CA, USA). Annexin V-FITC apoptosis detection kit was purchased from Vazyme (Nanjing, China). Nissl staining kit was purchased from Keygen Biotech (Nanjing, China). Antibodies of Bcl-2 (#3498), Bax (#2772), and cleaved caspase-3 (#14220) were purchased from Cell Signaling Technology Inc. (Danvers, MA, USA). αSyn antibody (#610787) was purchased from BD Biosciences (San Jose, CA, USA). Monoclonal anti phosphorylated αSyn antibody (pSyn#64; 015-25191) was purchased from Wako Pure Chemical Industries (Osaka, Japan). β-actin antibody (YFMA0052) was purchased from Yifeixue Biotech (Nanjing, China). 3, 3′-diaminobenzidine (DAB) kit was purchased from Beyotime (Shanghai, China).

### Purification of Recombinant Human Wild-Type αSyn and Preparation of αSyn PFF

Recombinant human wild-type αSyn was prepared and purified as previously described (Hua et al., 2017). The expression of monomeric αSyn-His was confirmed by Western analysis (Supplementary Figure 1A). According to the manufacturer's instruction, bacterial endotoxins were removed using the ToxinEraser™ Endotoxin Removal Kit. The endotoxin level was determined using the ToxinSensor™ Chromogenic LAL Endotoxin Assay Kit. Endotoxin-free αSyn contains less than 0.12 EU/ml of endotoxin. The concentration of monomeric αSyn was prepared above to 5 mg/ml with sterile phosphate buffer saline (PBS) and shook at 37°C, 1,000 rpm for seven days. The liquid containing the αSyn monomers appeared turbid after fibril formation (Supplementary Figure 1B). Sonicated αSyn-PFF was prepared via sonicating six times before each experiment (total 10 s, 1 s on, 1 s off). Thioflavin T (ThT) assay (Supplementary Figure 1C), SDS-PAGE (Supplementary Figure 1D), and electron microscope (Supplementary Figure 1E) were used to identify αSyn morphologies before subsequent experiments. The majority of sonicated αSyn-PFF was less than 50 nm (Supplementary Figure 1F). The average length of αSyn-PFF was 41.96 ± 0.80 nm.

### Cell Culture and Treatment

Human SH-SY5Y neuroblastoma cells were cultured as previously described (Hua et al., 2017). The medium was replaced with a serum-free medium containing PNU-282987 or nicotine. After one hour, 1 μM αSyn-PFF or 500 μM MPP^+^ was added into the medium for 24 h. The selective α7-nAChRs antagonist MLA was added one hour before the addition of PNU-282987 or nicotine.

### MTT Assay and Lactate Dehydrogenase (LDH) Assays

The MTT assay and LDH-based cytotoxicity assays were used to measure cell death and survival, as previously described (Hua et al., 2017).

### Hoechst 33342 Staining

After washing with PBS three times and fixed in 4% paraformaldehyde for 30 min, SH-SY5Y cells were stained with Hoechst 33342 (10 μg/mL, 20 min). The cells with highly-condensed and brightly-stained nuclei under a fluorescence microscope (Zeiss AX10; Carl Zeiss Microscopy GmbH, Jena, Germany) were regarded as apoptotic cells. The apoptotic index was defined as the ratio of the apoptotic cell number to the total cell number.

### Flow Cytometry

According to the manufacturer's instructions, the apoptosis of SH-SY5Y cells was detected by Annexin V-FITC Apoptosis Detection kits. The SH-SY5Y cells treated with different drugs were trypsinized and washed three times with PBS. After centrifugation, the cells were resuspended in 500 μl binding buffer with 5 μl Annexin V-FITC plus 5 μl Propidium Iodide (PI). After mixed gently and reacted for 15 min in the dark at room temperature, samples were analyzed in a flow cytometer (Guava EasyCyte; Millipore, Billerica, MA, USA).

### JC-1 Staining

The dissipation of mitochondrial electrochemical potential gradient is an early apoptosis event (Tusskorn et al., 2019). The digested cells were washed three times with PBS, stained with JC-1 dye solution at 37°C for 30 min, and finally detected the fluorescence intensity of the cells with a Guava EasyCyte flow cytometer to analyze the mitochondrial membrane potential.

### Western Blot Analysis

In general, the cells were lysed with RIPA lysis buffer (50 mM Tris-HCl, pH 7.4, 150 mM NaCl, 1% Nonidet P-40, 1 mM sodium orthovanadate) containing protease inhibitors and phosphatase inhibitors, and the proteins were extracted from the cells.

For detergent-solubility fractionation of phosphorylated αSyn (pS129-αSyn), the cells first were lysed with 1% Triton X-100 (TX-100) extraction buffer (50 mM Tris-HCl, pH 7.4, 150 mM NaCl, 1% Triton X-100) containing protease inhibitors and phosphatase inhibitors, sonicated, and centrifuged at 100,000 g for 30 min. The supernatant was collected as the Triton X-100 - soluble fraction. Then, the pellet was dissolved in 2% SDS RIPA buffer (50 mM Tris-HCl, pH 7.4, 150 mM NaCl, 1% Nonidet P-40, 1 mM sodium orthovanadate, and 1% SDS). The supernatant was collected as the SDS soluble fraction.

The proteins were denatured in SDS sample buffer, separated by 10% SDS-PAGE, and transferred to PVDF membranes. Blots were blocked with 5 % skimmed milk in Tris-buffered saline with 0.1 % Tween 20 (TBST) for 1 h and then washed three times with TBST buffer for 10 min each. The blots were then incubated with the following primary antibodies: Bcl-2 (1:1000), Bax (1:1000), cleaved caspase-3 (1:1000), αSyn (1:500), pS129-αSyn (1:200), or β-actin (1:10000) overnight at 4°C. The membranes were washed four times with TBST for 10 min each time, then incubated the secondary antibody, and washed four times with TBST. Finally, the membrane was observed and analyzed with Tanon 5200 (Tanon Science and Technology Co. Ltd, Shanghai, China).

### Construction and Culture of αSyn Stable Transgenic Cell Line

To generate a stable transgenic cell line expressing the human wild-type αSyn, SH-SY5Y cells were transfected with lentivectors pLenti-EF1a-EGFP-F2A-Puro-CMV-SNCA-Myc-His or pLenti-EF1α-EGFP-F2A-Puro-CMV-MCS according to the procedure recommended by the manufacturer (Obio Technology, Shanghai, China). The stable transfectants were selected in 2 μg/ml puromycin and further maintained in 1 μg/ml puromycin added to SH-SY5Y growth media.

### Animals and Treatment

Alpha7-nAChRs KO mice (male, 10-12 weeks old, weighing 24-28 g, C57BL/6J background) were purchased from the Jackson Laboratory (B6.129S7-charna7tm1bay, number 003232; Bar Harbor, ME, USA). Wild-type (WT) C57BL/6J mice (male, 10-12 weeks old, weighing 24-28 g) were purchased from the Animal Core Facility of Nanjing Medical University (Nanjing, Jiangsu, China). The mice were fed standard rodent food and water ad libitum. The room temperature was maintained at 24 ± 2°C, and the daily light and dark time were 12 h each. All animal experiments were approved by the Institutional Animal Care and Use Committee of Nanjing Medical University (IACUC).

For the chronic MPTP regimen, KO and WT mice received ten doses of MPTP (20 mg/kg in saline, *s.c*.) with probenecid (250 mg/kg *i.p*., twice a week for 5 weeks) as previously described (Sun et al., 2016). The mice in the control group were treated with normal saline and the same dose of probenecid. The mice were sacrificed 3.5 days after the first injection and 7 days after the last injection (42 days after the first injection).

### Nissl Staining and Immunohistochemistry

After mice were perfused with 4% paraformaldehyde, brains were dissected out, maintained in 4% paraformaldehyde overnight, and then cryopreserved in 30% sucrose in PBS and stored at −70°C until used. Twenty-micrometer coronal sections were cut and stained with Nissl according to the instructions. The sections were incubated with anti-TH antibody (1:4000) or anti-αSyn antibody (1:200) overnight at 4°C, and then incubated with HRP conjugated secondary antibodies (1:10000, Beyotime, Shanghai, China) at room temperature for 1 h. Finally, the brain slices were stained in a solution of DAB according to the manufacturer's instructions.

### Stereology

As previously reported (Sun et al., 2016), the numbers of Nissl, TH, and αSyn positive cells in the substantia nigra pars compacta (SNpc) were assessed using an unbiased stereological procedure with an optical fractionator (MicroBrightField Inc., Williston, VT). The regions of SNpc in the sections were outlined at low magnification (40 ×). The counting frame size was 40 × 40 μm, and the sampling grid size was 80 × 80 μm. The sampling scheme was designed to have a coefficient of error (CE) less than 10 % to get reliable results. All stereological analyses were carried out under the 200 × objective of an Olympus BX52 microscope (Olympus America Inc., Melville, NY).

### Statistical Analysis

All data were expressed as mean ± S.E.M. Statistical analysis was performed by two-way or one-way analysis of variance (ANOVA) followed by Tukey *post-hoc* test using GraphPad Prism software (San Diego, CA, USA). *Post-hoc* power calculations were performed G^*^Power 3.1.9.7 software with α = 0.05. *P* < 0.05 was considered to be statistically significant.

## Results

### Activation of α7-nAChRs Alleviated αSyn-PFF-Induced SH-SY5Y Cells Damage

**Figure 1:**
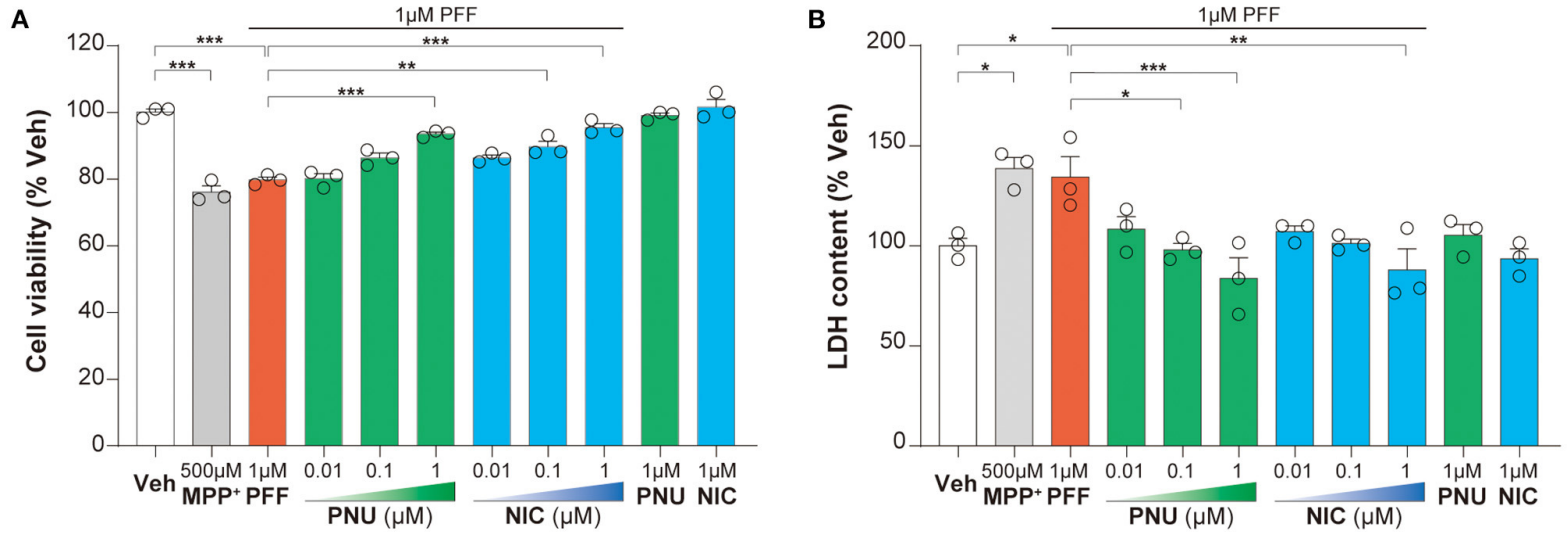
Activation of α7-nAChRs alleviated the αSyn-PFF-induced damage of SH-SY5Y cells. Pretreatment with PNU-282987 (PNU; 0.01, 0.1, and 1 μM) and nicotine (NIC; 0.01, 0.1, and 1 μM) for one hour protected against cell damage caused by αSyn-PFF (PFF). Cell viability was measured using the MTT assay **(A)**, whereas the level of cell death was determined by LDH assay **(B)**. Data were mean ± SEM for three independent experiments with three multiple holes in each group. *P < 0.05, **P < 0.01, and ***P < 0.001.

To determine the involvement of α7-nAChRs in αSyn pathology, we investigated the effects of PNU-289287 and nicotine on αSyn-PFF-induced cell injury. In SH-SY5Y cells, 1 μM αSyn-PFF produced a reduction (79.8 ± 0.8 %, *P* < 0.001) in cell proliferation by MPP^+^ used a positive control (76.1 ± 1.8 %, *P* < 0.001), while 1 μM PNU-282987 or 1 μM nicotine alone did not affect the cell proliferation (Figure 1A). Pretreatment with PNU-282987 (0.01 μM: 80.2 ± 1.4 %, *p* > 0.999; 0.1 μM: 86.5 ± 1.3 %, *p* = 0.0526; 1 μM: 93.6 ± 0.6 %, *P* < 0.0001) or nicotine (0.01 μM: 86.4 ± 0.8 %, *p* = 0.0573; 0.1 μM: 89.8 ± 1.7 %, *p* = 0.0010; 1 μM: 95.5 ± 1.2 %, *P* < 0.0001) significantly enhanced cell viability in a dose-related manner compared to the αSyn-PFF group (Figure 1A). *Post-hoc* analysis indicated that this experiment had a power of 90.6 % to detect a significant difference among the groups.

The increased leakage of LDH from cells into the medium indicates cell death. In SH-SY5Y cells, αSyn-PFF (134.3 ± 10.2%, *p* = 0.0398) and MPP^+^ (138.6 ± 5.5%, *p* = 0.0145) increased LDH leakage compared to the control cells, while pretreatment with PNU-282987 (0.01 μM: 108.4 ± 6.3 %, *p* = 0.2267; 0.1 μM: 98.0 ± 3.2 %, *p* = 0.0251; 1 μM: 83.7 ± 10.4 %, *p* = 0.0008) or nicotine (0.01 μM: 107.2 ± 2.8 %, *p* = 0.1816; 0.1 μM: 101.2 ± 2.1%, *p* = 0.0522; 1 μM: 88.4 ±10.38 %, *p* = 0.0022) attenuated this LDH leakage in a dose-related manner compared to the αSyn-PFF group (Figure 1B). There was no significant difference in the leakage of LDH with 1 μM PNU-282987 or 1 μM nicotine alone. *Post-hoc* analysis indicated that this experiment had a power of 81.1 % to detect a significant difference among the groups.

These results showed that α7-nAChRs activation could alleviate the αSyn-PFF-induced damage of SH-SY5Y cells.

### Activation of α7-nAChRs Reversed αSyn-PFF-Induced Apoptosis in SH-SY5Y Cells

**Figure 2:**
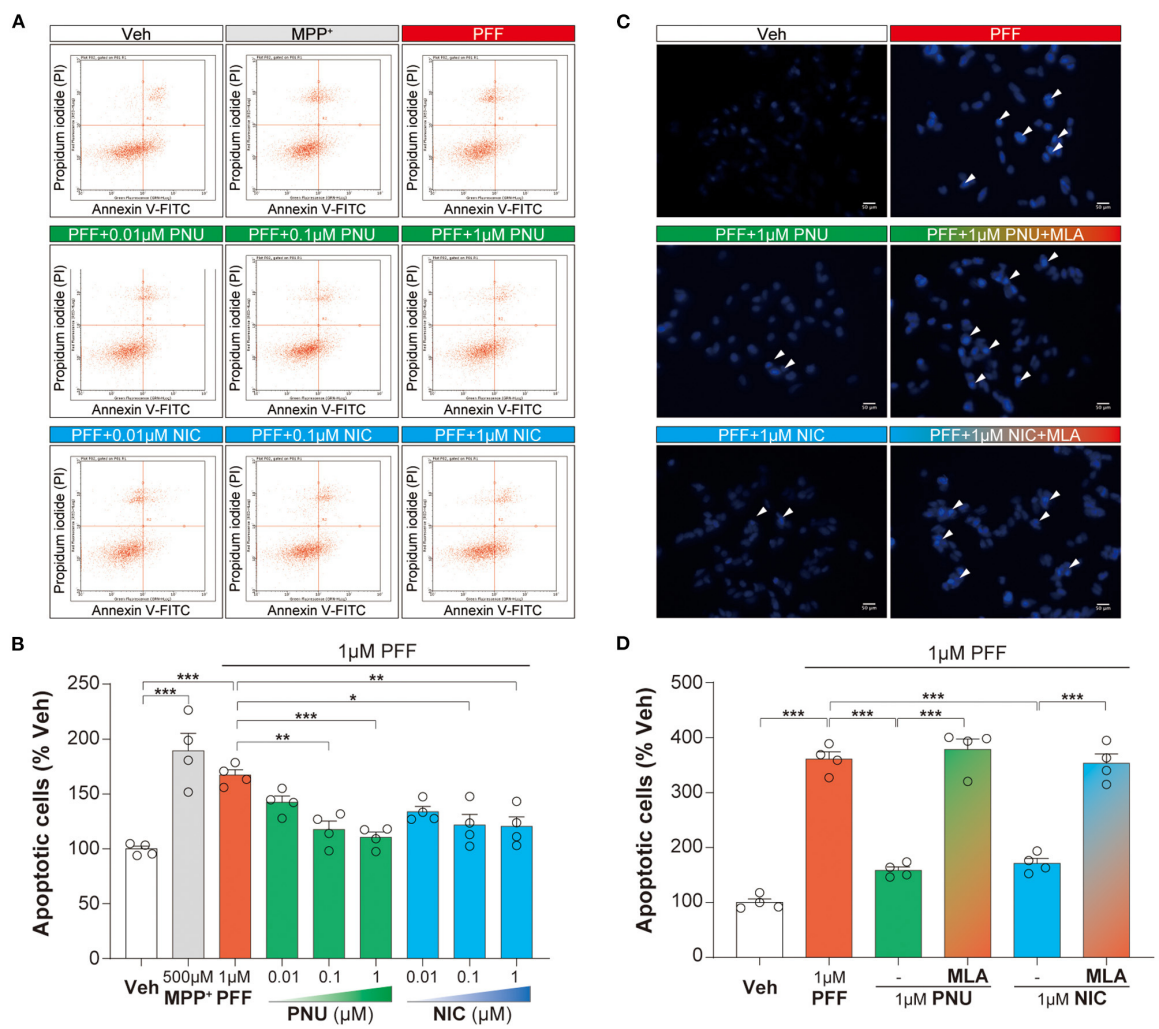
Activation of α7-nAChRs alleviated the αSyn-PFF-induced apoptosis of SH-SY5Y cells. Pretreatment with PNU-282987 (PNU; 0.01, 0.1, and 1 μM) and nicotine (NIC; 0.01, 0.1, and 1 μM) for one hour inhibited cell apoptosis caused by αSyn-PFF (PFF). Apoptotic cells were tested by flow cytometry using FITC-labeled annexin (Annexin V-FITC) and pyridinium iodide **(A,B)**. Data were mean ± SEM for four independent experiments with three multiple holes in each group. *P < 0.05, **P < 0.01, and ***P < 0.001. Quantification for analysis of cells with apoptosis was using fluorescent staining with Hoechst 33342 **(C,D)** when adding the selective α7-nAChRs antagonist MLA one hour before the addition of PNU-282987 or nicotine. Scale bars: 50 μm. Data were mean ± SEM for four independent experiments. ***P < 0.001.

Since apoptosis plays a vital role in the neurotoxicity of αSyn, we calculated the αSyn-PFF-induced apoptotic changes with PNU-282987 and nicotine. A flow cytometric analysis for apoptosis indicated that 1 μM αSyn-PFF caused significant apoptosis (167.1 ± 4.8 %, *P* < 0.0001), which was reversed in a dose-related manner by pretreated with PNU-282987 (0.01 μM: 142.4 ± 5.6 %, *p* = 0.4403; 0.1 μM: 117.7 ± 7.5 %, *p* = 0.0044; 1 μM: 110.5 ± 4.8 %, *p* = 0.0009) or nicotine (0.01 μM: 133.8 ± 4.7 %, *p* = 0.1207; 0.1 μM: 121.6 ± 9.7 %, *p* = 0.0105; 1 μM: 120.3 ± 8.8 %, *p* = 0.0078, Figures 2A,B). *Post-hoc* analysis indicated that this experiment had a power of 91.9 % to detect a significant difference among the groups. Then, 1 μM PNU-282987 and 1 μM nicotine were chosen for the following experiment.

Similarly, Hoechst 33342 staining assay revealed that 1 μM αSyn-PFF increased the rate of apoptotic cells (361.1 ± 12.9 %, *P* < 0.0001) compared to the control group. When pretreated with 1 μM PNU-282987 (158.6 ± 6.3 %, *P* < 0.0001) and 1 μM nicotine (171.4 ± 8.9 %, *P* < 0.0001), the rate of apoptotic cells was significantly reduced (Figures 2C,D). These anti-apoptotic effects of α7-nAChRs activation were expectedly reversed by pretreatment with 100 nM MLA (PNU-282987 + MLA: 378.1 ± 19.3 %, *P* < 0.0001; nicotine + MLA: 353.2 ± 17.1 %, *P* < 0.0001; Figures 2C,D). *Post-hoc* analysis indicated that this experiment had a power of 83.1 % to detect a significant difference among the groups. These findings confirmed that α7-nAChRs activation produced anti-apoptotic effects on αSyn-PFF-induced neurotoxicity.

### Activation of α7-nAChRs Inhibited αSyn-PFF-Induced Mitochondrial Apoptosis in SH-SY5Y Cells

**Figure 3:**
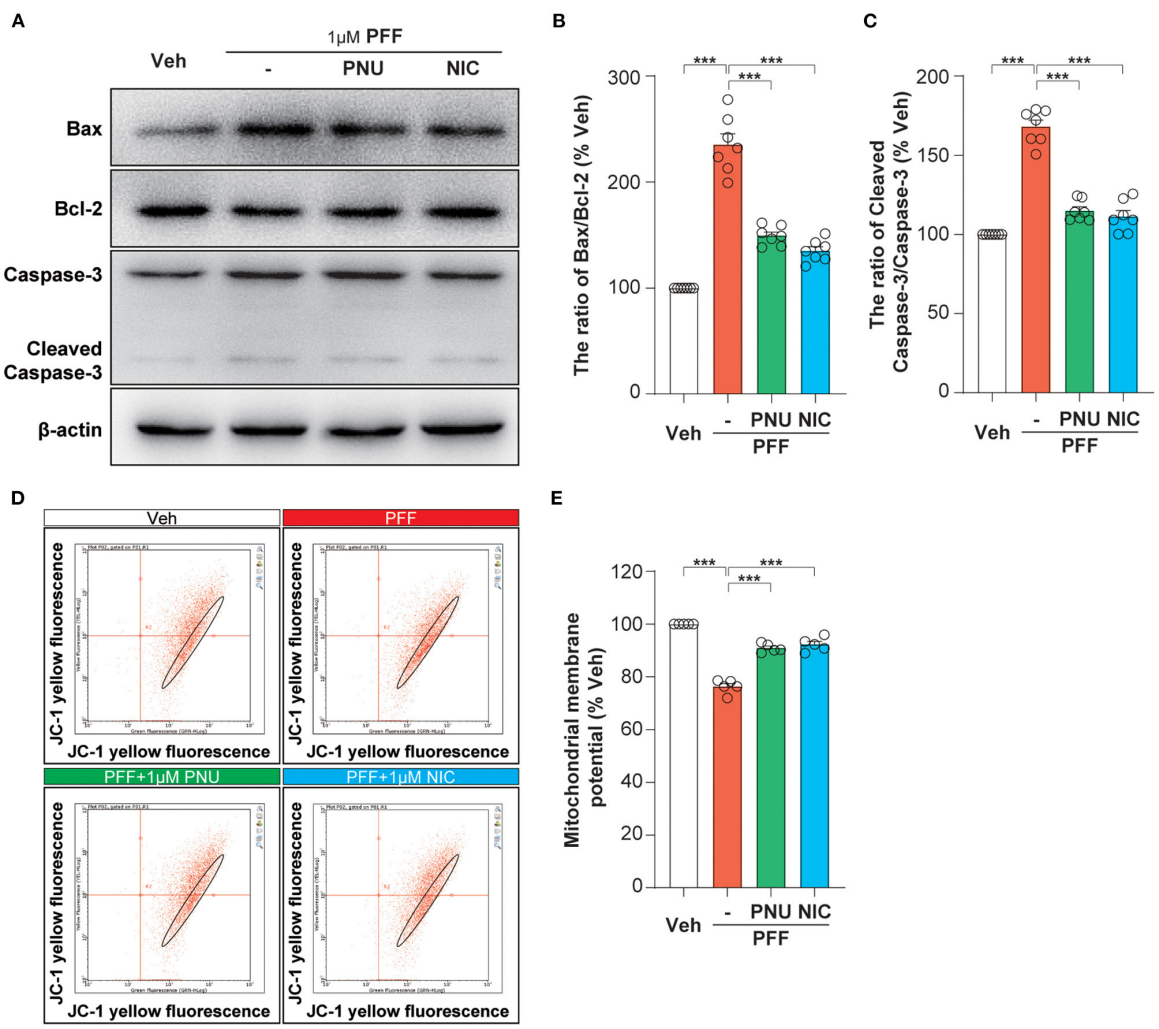
Activation of α7-nAChRs inhibited αSyn-PFF-induced mitochondrial apoptosis of SH-SY5Y cells. Pretreatment with PNU-282987 (PNU) and nicotine (NIC) for one hour inhibited mitochondrial apoptosis caused by αSyn-PFF (PFF). Representative blots were shown in **(A)**. The Bax/Bcl-2 ratio **(B)** and cleaved-Caspase-3/Caspase-3 ratio **(C)** were measured by Western blot analysis. Data were mean ± SEM for seven independent experiments. ***P < 0.001. Evaluation of mitochondrial membrane potential stained with bivariate JC-1 dye **(D)** in SH-SY5Y cells by flow cytometry. Quantification of mitochondrial membrane potential (% of the vehicle) was expressed as a JC-1 ratio **(E)**. Data were mean ± SEM for five independent experiments. *p < 0.05 and ***p < 0.001.

To examine the expression levels of apoptosis-associated proteins exposed to αSyn-PFF, we observed the levels of Bax, Bcl-2, and cleaved-caspase-3 analyzed by Western blot assay (Figure 3A). The treatment of αSyn-PFF stimulated the ratio of Bax/Bcl-2 (235.3 ± 10.2 %, *P* < 0.0001, Figure 3B) and the expression of cleaved-caspase-3 (168.1 ± 4.1 %, *P* < 0.0001, Figure 3C), compared to the control group. Pretreatment with PNU-282987 or nicotine inhibited the αSyn-PFF-induced alterations of Bax/Bcl-2 ratio (PNU-282987: 149.4 ± 3.4 %, *P* < 0.0001; nicotine: 135.0 ± 3.9 %, *P* < 0.0001; Figure 3B) and cleaved-caspase-3 (PNU-282987: 114.9 ± 2.5 %, *P* < 0.0001; nicotine: 111.4 ± 3.7 %, *P* < 0.0001; Figure 3C). *Post-hoc* analysis indicated that these experiments had a power of 86.2 % for Bax/Bcl-2 ratio and a power of 73.0 % for cleaved-caspase-3 to detect a significant difference among the groups.

Bcl-2 and Bax are potent regulators of apoptosis via regulating the permeability of the mitochondrial membrane. The αSyn-PFF-induced alteration of the mitochondrial membrane potential was further determined by the molecular probe JC-1 (Figure 3D). Bivariate analysis of mitochondrial membrane potential showed that a loss of the mitochondrial membrane potential (76.3 ± 1.2 %, *P* < 0.0001) in αSyn-PFF-treated cells was alleviated by the pretreatment with PNU-282987 (90.9 ± 0.7 %, *P* < 0.0001) or nicotine (92.3 ± 1.2 %, *P* < 0.0001; Figure 3E). *Post-hoc* analysis indicated that these experiments had a power of 90.3 % to detect a significant difference among the groups. We conclude that α7-nAChR stimulation contributes to the limitation of the mitochondrial pathway of apoptosis induced by αSyn-PFF.

### Activation of α7-nAChRs Suppressed the Accumulation of αSyn in SH-SY5Y Cells and Stable Transgenic Cell Line

**Figure 4:**
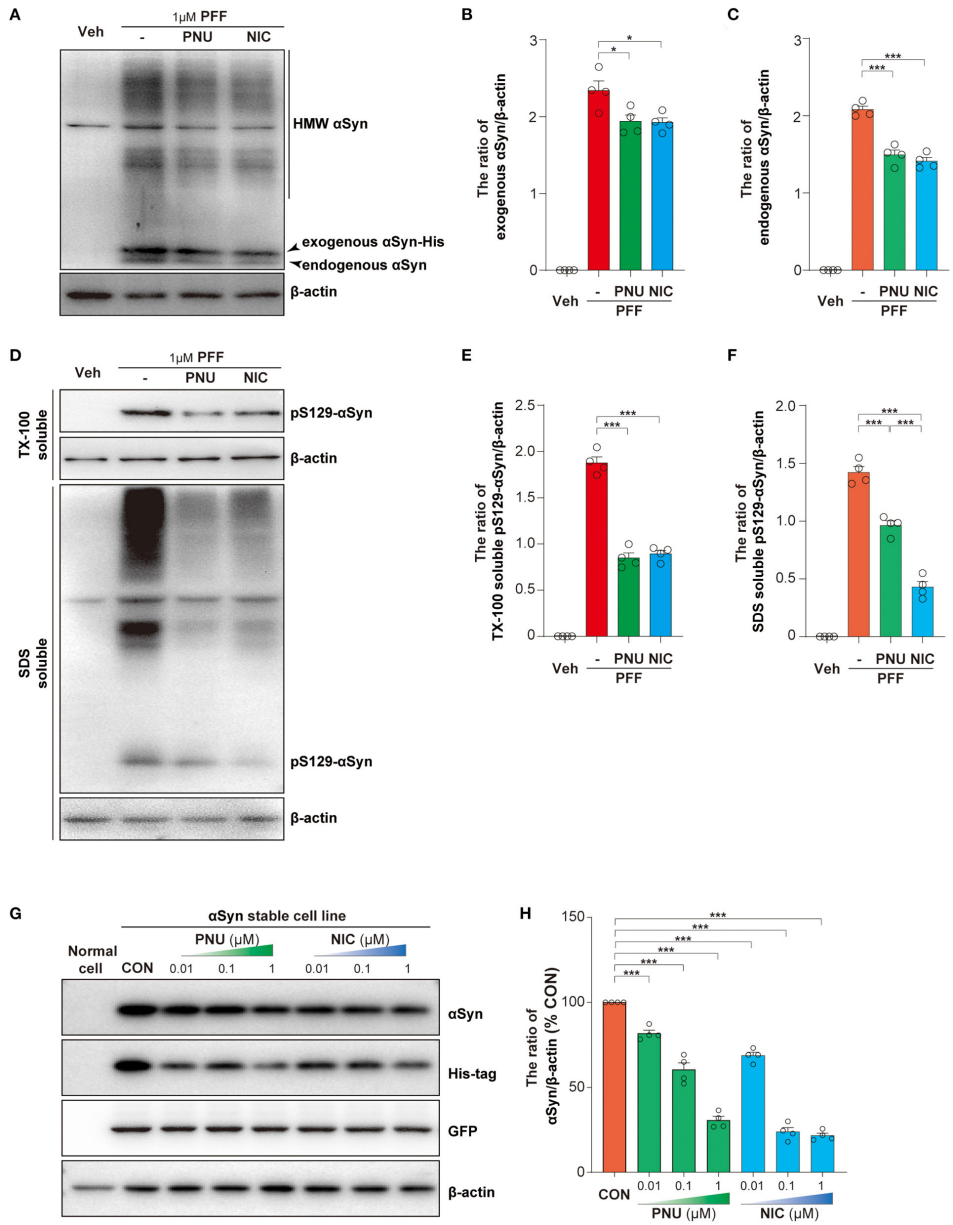
Activation of α7-nAChRs reduced the levels of intracellular αSyn. Representative immunoblots of αSyn in SH-SY5Y cells treated with αSyn-PFF **(A)**. Treatment with PNU-282987 (PNU) and nicotine (NIC) inhibited the increased intracellular αSyn caused by αSyn-PFF (PFF), including exogenous αSyn **(B)** and endogenous αSyn **(C)**. Representative immunoblots of phosphorylated αSyn (pS129-αSyn) in SH-SY5Y cells treated with αSyn-PFF **(D)**. Treatment with PNU-282987 (PNU) and nicotine (NIC) inhibited the phosphorylation of αSyn caused by αSyn-PFF (PFF), including in the Triton X-100 (TX-100)-soluble fraction **(E)** and the SDS—soluble fraction **(F)**. Representative immunoblots of αSyn, His-tag, and GFP after the treatment of PNU or NIC **(G)**. Quantification of αSyn (% of the non-treated stable cell line) was expressed as an αSyn/β-actin ratio **(H)**. Data were mean ± SEM for four independent experiments. *p < 0.05 and ***p < 0.001.

To further explore the protective mechanism of α7-nAChRs on cell apoptosis, we first calculated the levels of intracellular αSyn. As previously reported (Luk et al., 2009; Apetri et al., 2016), αSyn-PFF exogenously added was taken up by SH-SY5Y cells and then induced the increase of endogenous αSyn (Figure 4A). Interestingly, compared with αSyn-PFF alone (exogenous: 2.34 ± 0.12; endogenous: 2.08 ± 0.04), the pretreatment of PNU-282987 or nicotine led to fewer amounts of HMW exogenous αSyn with a concomitant reduction of both monomeric exogenous αSyn (PNU-282987: 1.94 ± 0.08, *p* = 0.0185; nicotine: 1.93 ± 0.06, *p* = 0.0149; Figure 4B) and endogenous αSyn (PNU-282987:1.49 ± 0.06, *P* < 0.0001; nicotine: 1.41 ± 0.05, *P* < 0.0001; Figure 4C). *Post-hoc* analysis indicated that these experiments had a power of 78.8 % for exogenous αSyn and 78.6 % for endogenous αSyn to detect a significant difference among the groups.

Phosphorylation of αSyn at serine-129 is thought to be a marker of αSyn pathology. As showed in Figure 4D, αSyn-PFF exogenously induced the increase of pS129-αSyn in the TX-100 - soluble fraction (1.88 ± 0.06) and the SDS - soluble fraction (1.42 ± 0.05). the pretreatment of PNU-282987 or nicotine inhibited the phosphorylation of αSyn in the TX-100—soluble fraction (PNU-282987: 0.85 ± 0.05, *P* < 0.0001; nicotine: 0.89 ± 0.04, *P* < 0.0001; Figure 4E) and the SDS - soluble fraction (PNU-282987:0.96 ± 0.04, *P* < 0.0001; nicotine: 0.43 ± 0.05, *P* < 0.0001; Figure 4F). *Post-hoc* analysis indicated that these experiments had a power of 78.9 % for αSyn in the TX-100—soluble fraction and 78.8 % for αSyn in the SDS - soluble fraction to detect a significant difference among the groups.

We next established an αSyn stable transgenic cell line and examined whether α7-nAChRs activation promoted the clearance of αSyn species. The stable transgenic cell line containing a non-fusion GFP and a C-terminal Myc-His-tag was confirmed by Western blotting analysis (Supplementary Figure 2A) and immunofluorescence (Supplementary Figure 2B). We confirmed that 1 μM PNU-282987 (Supplementary Figure 2C) or 1 μM nicotine (Supplementary Figure 2D) alone did not affect cell proliferation for 24 h or 48 h of treatment. Both PNU-282987 and nicotine significantly reduced αSyn and His-tag expressions, not GFP expression (Figure 4G). Compared with non-treated stable transgenic cell line, PNU-282987 (0.01 μM: 81.8 ± 1.9 %, *p* = 0.0.002; 0.1 μM: 60.5 ± 4.0 %, *P* < 0.0001; 1 μM: 30.6 ± 2.3 %, *P* < 0.0001) and nicotine (0.01 μM: 68.8 ± 2.0 %, *P* < 0.0001; 0.1 μM: 23.8 ± 2.5 %, *P* < 0.0001; 1 μM: 21.6 ± 1.5%, *P* < 0.0001) reduced αSyn expressions in a dose-dependent manner (Figure 4H). *Post-hoc* analysis indicated that these experiments had a power of 93.8 % for αSyn expressions to detect a significant difference among the groups. These results indicate that α7-nAChRs activation may directly accelerate the clearance of αSyn.

### α7-nAChRs Deficiency Aggravated the Loss of Dopamine Neurons and Increased the Deposition of αSyn in a Chronic MPTP Mouse Model of PD

**Figure 5:**
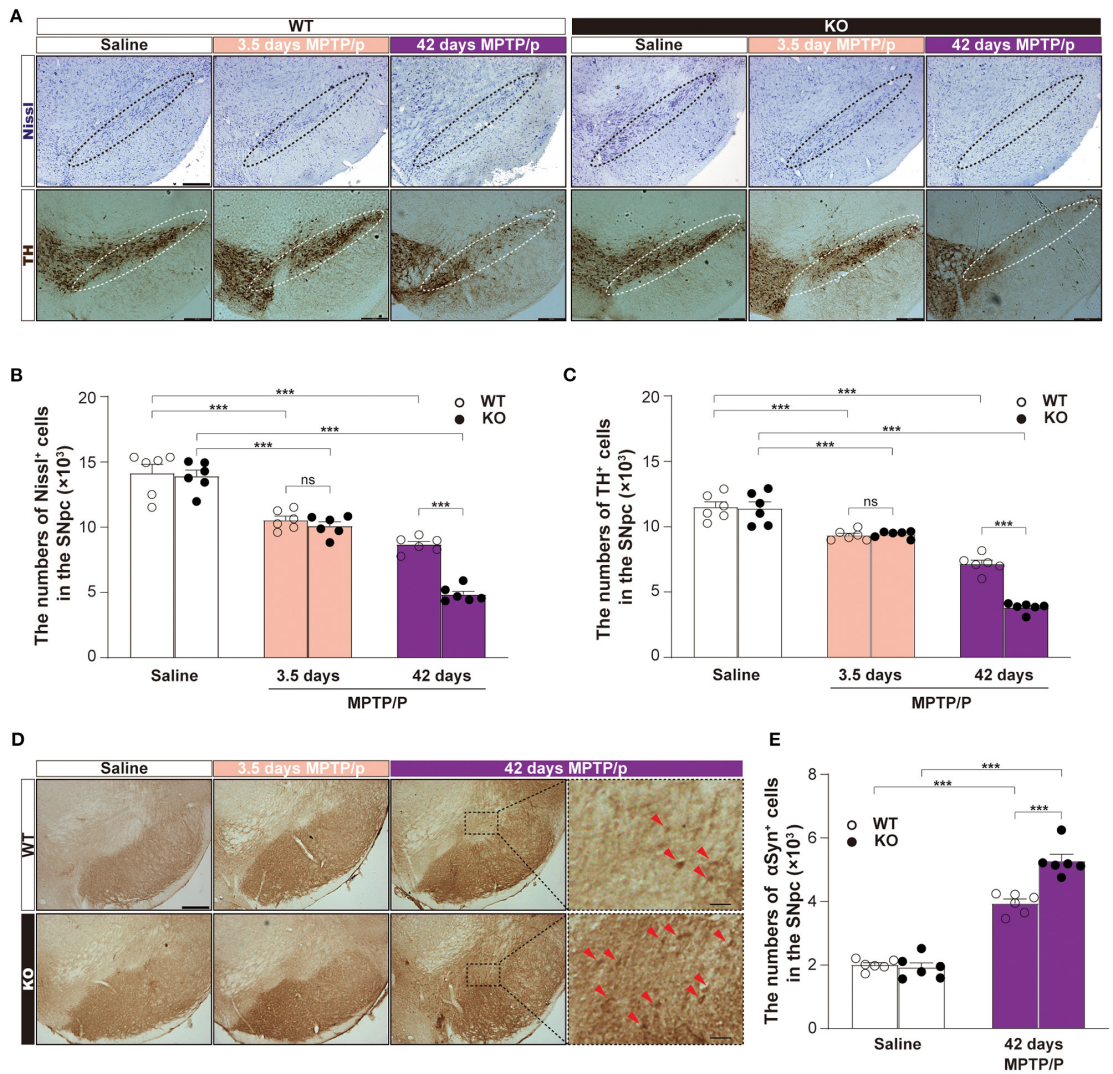
α7-nAChRs deficiency aggravated the loss of dopamine neurons and increased the deposition of αSyn in a chronic MPTP mouse model of PD. Representative pictures for Nissl-positive staining and TH-positive staining **(A)** were shown. Scale bar, 250 μm. Nonbiased stereological analysis of Nissl-positive staining cells **(B)** and TH-positive staining **(C)** in the SNpc from saline- and MPTP/p-treated α7-nAChRs knockout (KO) and wild-type (WT) mice. Data were mean ± SEM obtained with six mice of each group. ***P < 0.001. Representative pictures for αSyn-positive staining cells in the midbrain **(D)**. Scale bar, 250 μm. Forty-two days MPTP/p enlarged show the enlargement of αSyn-positive staining cells (red arrow) in SNpc. Scale bar, 50 μm. Nonbiased stereological analysis **(E)** of αSyn-positive staining cells in the SNpc from saline- and MPTP/p-treated α7-nAChRs KO and WT mice. Data were mean ± SEM obtained with six mice of each group. ***P < 0.001. MPTP/p: 1-methyl-4-phenyl-1, 2, 3, 6-tetrahydropyridine/probenecid; SNpc, substantia nigra pars compacta.

To confirm the potential role of α7-nAChRs in αSyn pathology and clearance *in vivo*, we established the chronic MPTP mouse models of PD using α7-nAChRs KO mice and WT mice. According to the immunohistochemical staining of the midbrain (Figure 5A) and stereological counting, there was no difference in the numbers of Nissl-positive neurons (KO: 13905 ± 463, WT: 14132 ± 684, *p* = 0.9717; Figure 5B) and TH-positive neurons (KO: 11399 ± 501, WT: 11495 ± 402, *p* = 0.5927; Figure 5C) in the SNpc between KO and WT mice. However, MPTP treatment markedly decreased the numbers of Nissl-positive neurons [Two-way ANOVA, genotype: *F*_1,30_ = 20.33, *P* < 0.0001; Treatment: *F*_2,30_ = 159.8, *P* < 0.0001; interaction: *F*_2,30_ = 12.22, *p* = 0.0001] and TH-positive neurons [Two-way ANOVA, genotype: *F*_1,30_ = 20.45, *P* < 0.0001; Treatment: *F*_2,30_ = 199.4, *P* < 0.0001; interaction: *F*_2,30_ = 20.14, *P* < 0.0001]. Interestingly, the reduced numbers of Nissl-positive neurons (10542 ± 303, *p* = 0.8255) and TH-positive neurons (9419 ± 104, *p* = 0.9971) in the SNpc of KO mice showed no difference than that of WT mice (Nissl: 10093 ± 313, TH: 9342 ± 159) 3.5 days after the first injection. Then, α7-nAChRs deficiency aggravated the loss of Nissl-positive neurons (KO: 4857 ± 243, WT: 8665 ± 240, *P* < 0.0001) and TH-positive neurons (KO: 3817 ± 150, WT: 7156 ± 283, *P* < 0.0001) 42 days after the first injection. Moreover, the chronic MPTP/p treatment increased the deposition of αSyn in the midbrain 42 days after the first injection (Figure 5D). Unlike 3.5 days after the first injection, the mice in 42 days developed αSyn-positive staining in the SNpc. Compared with matched WT mice (3944 ± 132), KO mice showed more αSyn-positive staining (5279 ± 206, *P* < 0.001) in the SNpc [Two-way ANOVA, genotype: *F*_1,20_ = 18.15, *p* = 0.0001; Treatment: *F*_1,20_ = 325.2, *P* < 0.0001; interaction: *F*_1,20_ = 23.34, *p* = 0.0001; Figure 5E]. These results indicate that α7-nAChRs play an important role in the process of MPTP-induced midbrain neuron loss via regulating the clearance of αSyn *in vivo*.

## Discussion

Numerous studies have shown the neuroprotective effects of nicotine in the PD model through α7-nAChRs (Kawamata and Shimohama, 2011; Nicholatos et al., 2018). In particular, *in vitro* and *in vivo* studies have shown that nicotine can strongly prevent neurotoxicity induced by selective toxic agents such as rotenone, 6-OHDA, and MPTP by activating α7-nAChRs expressed on DA neurons (Kawamata and Shimohama, 2011). Previous studies in our laboratory have also shown that the use of nicotine and PNU-282987 can inhibit MPP^+^-induced apoptosis of SH-SY5Y cells through the ERK/p53 signaling pathway (Xu et al., 2019). Here, using the toxic αSyn-PFF to model SH-SY5Y cells, we at first revealed that α7-nAChRs activation could significantly reduce the cytotoxicity of αSyn oligomeric species by inhibiting the mitochondrial apoptosis pathway. Then, we found an additive effect of α7-nAChRs on the clearance of αSyn *in vitro* and *in vivo*. Thus, we demonstrated for the first time that α7-nAChRs exhibited a neuroprotective effect on αSyn pathology and aggregation via promoting αSyn clearance.

Pathological αSyn is reported to trigger apoptosis in various cell types, especially mitochondria-dependent apoptosis (Yasuda and Mochizuki, 2010; Yasuda et al., 2013). Recent studies suggest that αSyn oligomers can localize to mitochondria, interact with ATP synthase, and open the mitochondria permeability transition pore (mPTP) (Ludtmann et al., 2018). The mPTP opening results in the depolarization of mitochondrial membrane potential and the activation of caspase-3, leading to apoptotic death. In agreement with these previous reports, our findings demonstrated increased cellular toxicity in SH-SY5Y cells upon treatment with extracellular αSyn-PFF. We previously documented an anti-apoptotic role of α7-nAChRs via regulating the ERK/p53 signaling pathway in MPP^+^-induced cell death models (Xu et al., 2019). Similarly, the α7-nAChRs agonist PNU-282987 and nicotine reduced apoptotic cell death induced by αSyn-PFF, indicating the α7-nAChRs-mediated neuroprotection both in MPP^+^- and αSyn-induced PD models. Activation of α7-nAChRs can inhibit the increased Bax/Bcl-2 ratio and cleaved-caspase-3, maintain mitochondrial membrane potential, and prevent αSyn-PFF-induced apoptosis. Thus, our findings confirm that α7-nAChRs are effective targets for protection against cell damage in the PD model. However, the protective mechanisms of α7-nAChRs on αSyn-PFF-induced apoptosis have not been elucidated.

Numerous studies found that the extracellular αSyn could internalize to the cells and enhance the intracellular formation of αSyn (Luk et al., 2009; Nonaka et al., 2010; Danzer et al., 2012). Pathogenic αSyn containing a mitochondrial targeting sequence is preferentially translocated and binds to mitochondria, inducing mitochondrial dysfunction and apoptosis (Wang et al., 2019). Our study revealed that exogenous αSyn-PFF could not only be taken up and accumulate in SH-SY5Y cells but induced the increase of endogenous αSyn and the phosphorylation of αSyn, which are consistent with findings from previous studies. Interestingly, we found that α7-nAChRs activation showed the reduction of both exogenous and endogenous αSyn, which was confirmed in αSyn stable transgenic cell line and a mouse model of PD using the chronic MPTP/P treatment. In addition to regulating dopaminergic neuronal death and apoptosis via MAPK signaling (Xu et al., 2019) or Wnt/β-catenin signaling pathways (Liu et al., 2017), our results indicate that α7-nAChRs play an important role in α-synucleinopathy through enhancing the clearance of αSyn.

Since smoking can reduce the incidence of PD, it is proposed whether nicotine affects the oligomerization and aggregation of αSyn. Some researchers found that nicotine *in vitro* could directly inhibit αSyn fibrillation, destabilize preformed αSyn fibrils, and stabilize soluble oligomeric αSyn (Ono et al., 2007; Hong et al., 2009). Using *in vitro* and a yeast model of PD, Kardani et al. (2017) also discovered that nicotine caused αSyn oligomerization inhibition. Recently, Federica Bono et al. (2019) showed that nicotine activated the dopamine D3R-nAChR complex and prevented αSyn accumulation in primary cultures of mouse dopaminergic neurons and human iPSC-derived dopaminergic neurons. However, chronic nicotine administration showed no effect on the levels of αSyn in the SNpc and striatum of Thy1-αSyn mice (Subramaniam et al., 2018). Besides, nicotine has intracellular machinery that governs protein folding, transport and turnover via regulating endoplasmic reticulum and mitochondrial health (Srinivasan et al., 2014). Thus, it is still controversial whether nicotine can modify the production, clearance, or aggregation of αSyn. In the present study, both PNU-282987 and nicotine dose-dependently attenuated the accumulation of αSyn after exposure to exogenous αSyn or overexpression of αSyn. Moreover, α7-nAChRs deficiency increased the deposition of αSyn in the SNpc and aggravated dopaminergic neurons' loss after the chronic MPTP/p treatment. Previous evidence for the impairment of cholinergic systems in PD patients and animal models, together with the current effect of α7-nAChRs agonists on αSyn accumulation, supports the hypothesis that α7-nAChRs is involved in regulating αSyn aggregation. Future studies in the PFF-induced mice model should determine whether chronic treatment of α7-nAChRs agonists can improve αSyn aggregation via direct activating this receptor.

There are many mechanisms for clearing neurotoxic proteins in the brain, such as the autophagy-lysosomal pathway (Wong and Cuervo, 2010), the chaperone-mediated autophagy (Djajadikerta et al., 2020), and the ubiquitin-proteasome system (Limanaqi et al., 2020). Among these, the ubiquitin-proteasome system is the main degradation pathway of αSyn under normal conditions, while the autophagy-lysosomal pathway is recruited with the increase of αSyn (Ebrahimi-Fakhari et al., 2011). It is interesting to note that, in a recent study using a mice model of inflammatory bowel disease, α7-nAChRs deficient mice showed an impairment of the autophagic function through reduction of AMPK-mTOR-p70S6K signaling pathway (Shao et al., 2019). Since we observed increased accumulation of αSyn in the SNpc of α7-nAChRs KO mice, activation of α7-nAChRs may promote αSyn clearance and degradation by enhancing the ubiquitin-proteasome system or the autophagy-lysosomal pathway. Additionally, we did not rule out the possibility that a7-nAChRs agonist directly decreased extracellular αSyn-PFF or blocks αSyn uptake in the present study. Thus, the exact mechanisms of α7-nAChRs on αSyn clearance are not very clear. They need more investigations, including the effects of α7-nAChRs on the ubiquitin-proteasome system and the autophagy-lysosomal pathway in the PFF-induced mice model.

## Conclusion

Our results found that α7-nAChRs activation protected against apoptosis induced by αSyn-PFF, partially via inhibiting αSyn accumulation and phosphorylation. Although the exact mechanisms of α7-nAChRs on αSyn clearance are not very clear and need more investigations, our study provides ideas for finding new targets for PD from the perspective of αSyn clearance.

## Data Availability Statement

The raw data supporting the conclusions of this article will be made available by the authors, without undue reservation.

## Ethics Statement

The animal study was reviewed and approved by the Institutional Animal Care and Use Committee of Nanjing Medical University.

## Author Contributions

YF, JZhang, and YH conceived and planned the experiments. JZhao, SX, and TT carried out cellular experiments. YuL and YaL designed and performed the animal experiment. JZhao and TT performed biochemical analysis. YH and JZhang contributed to the interpretation of the results. JZhang and YF wrote the manuscript. All authors reviewed and approved the final manuscript.

## Conflict of Interest

The authors declare that the research was conducted in the absence of any commercial or financial relationships that could be construed as a potential conflict of interest.
