# Supplementary figures and images for: Activation of α7-nAChRs Promotes the Clearance of α-Synuclein and Protects Against Apoptotic Cell Death Induced by Exogenous α-Synuclein Fibrils
Source: Front Cell Dev Biol. 2021 Feb 25;9:637319. doi: 10.3389/fcell.2021.637319 (PMC7947362; doi:10.3389/fcell.2021.637319)

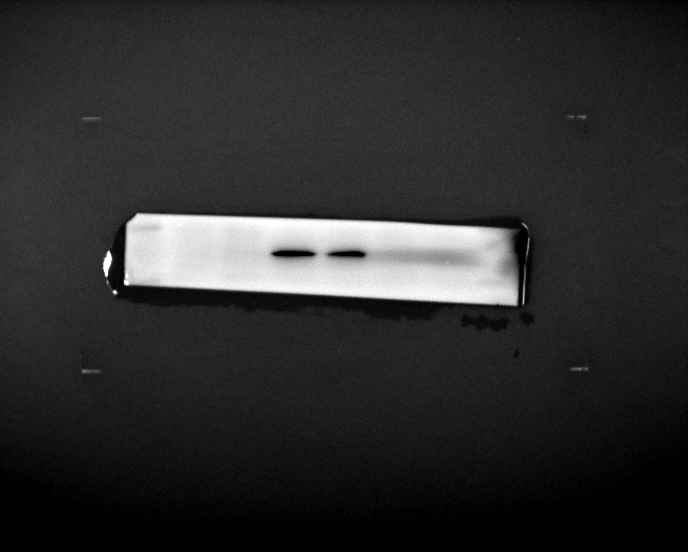

Supplement: Supplementary file 1 [file Data_Sheet_1.ZIP › spFig.2A.tif]

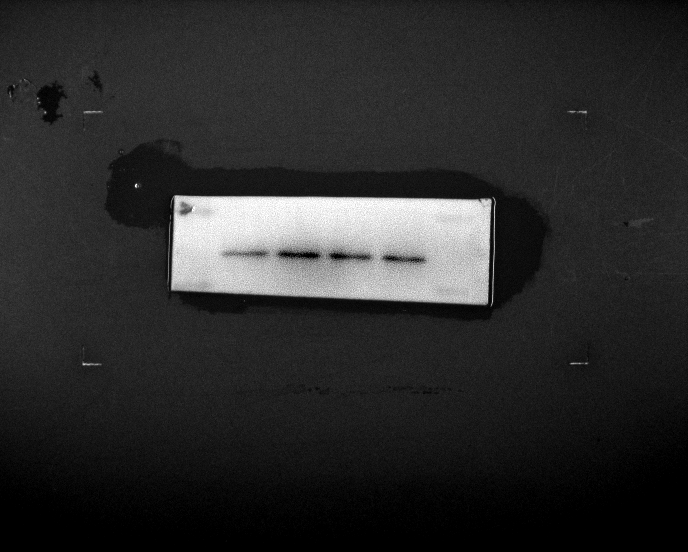

Supplement: Supplementary file 1 [file Data_Sheet_1.ZIP › Fig.3A Bax.tif]

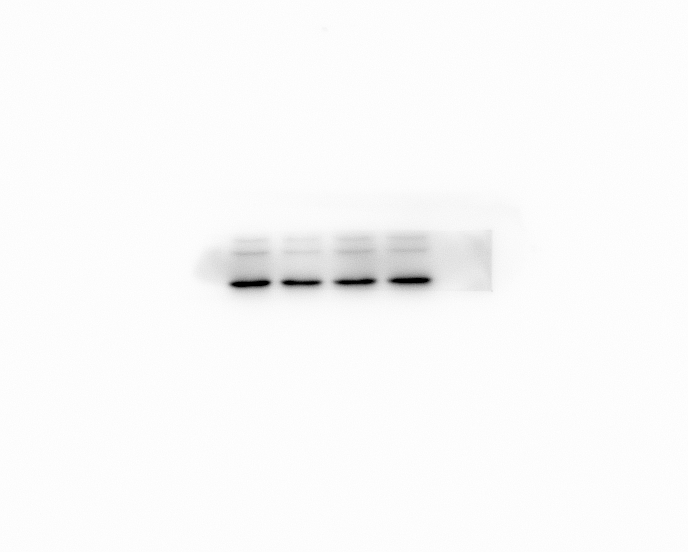

Supplement: Supplementary file 1 [file Data_Sheet_1.ZIP › Fig.3A Bcl-2.tif]

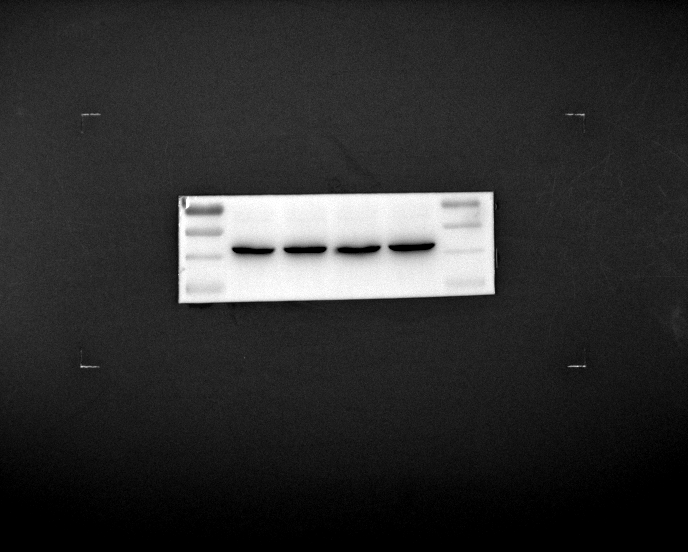

Supplement: Supplementary file 1 [file Data_Sheet_1.ZIP › Fig.3A beta-actin.tif]

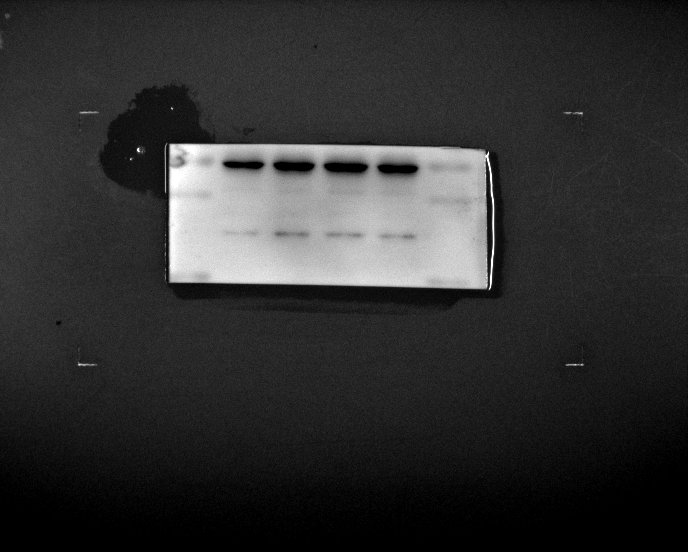

Supplement: Supplementary file 1 [file Data_Sheet_1.ZIP › Fig.3A caspase-3.tif]

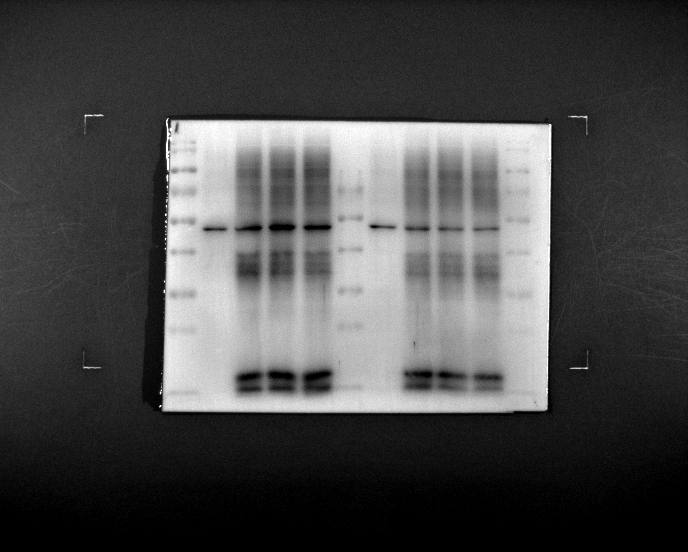

Supplement: Supplementary file 1 [file Data_Sheet_1.ZIP › Fig.4A aSyn.tif]

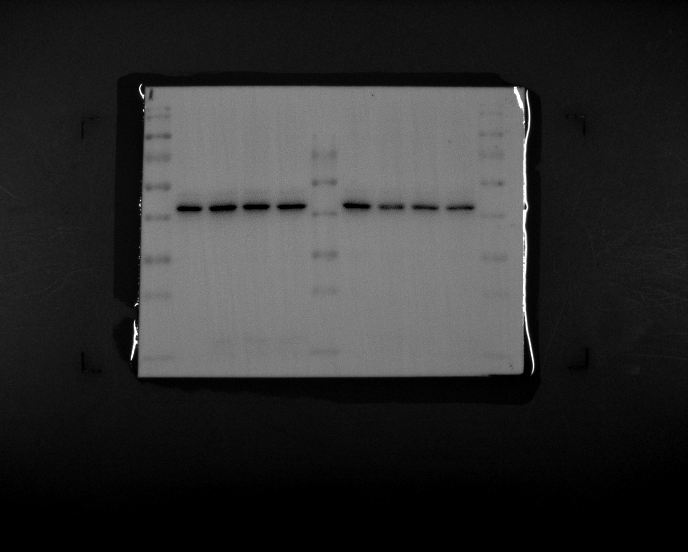

Supplement: Supplementary file 1 [file Data_Sheet_1.ZIP › Fig.4A beta-actin-New.tif]

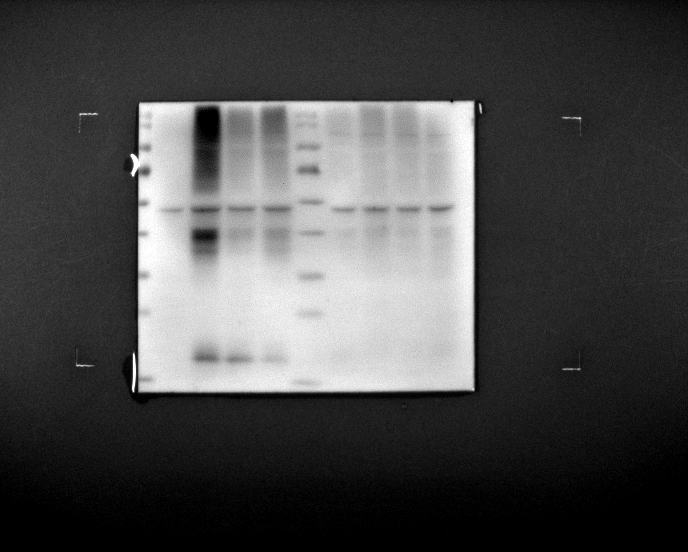

Supplement: Supplementary file 1 [file Data_Sheet_1.ZIP › Fig.4D SDS-aSyn.tif]

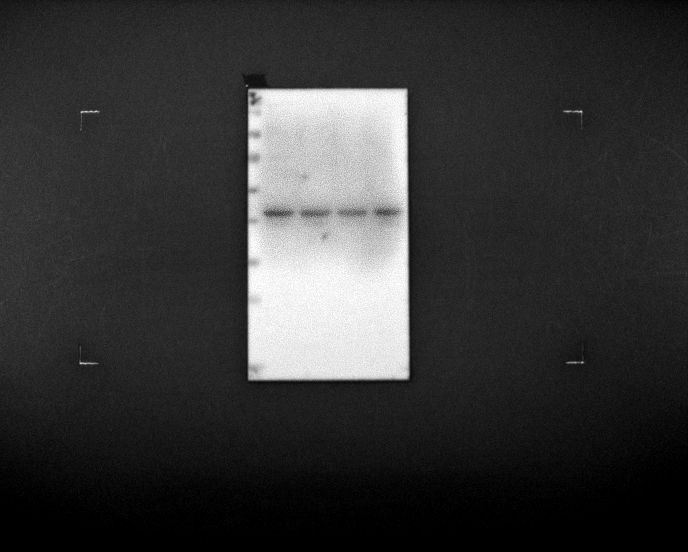

Supplement: Supplementary file 1 [file Data_Sheet_1.ZIP › Fig.4D SDS-beta-actin.tif]

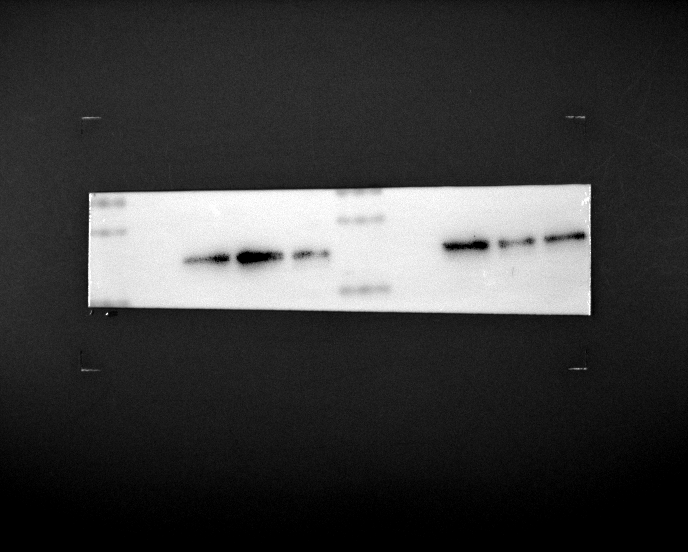

Supplement: Supplementary file 1 [file Data_Sheet_1.ZIP › Fig.4D TX-100-aSyn.tif]

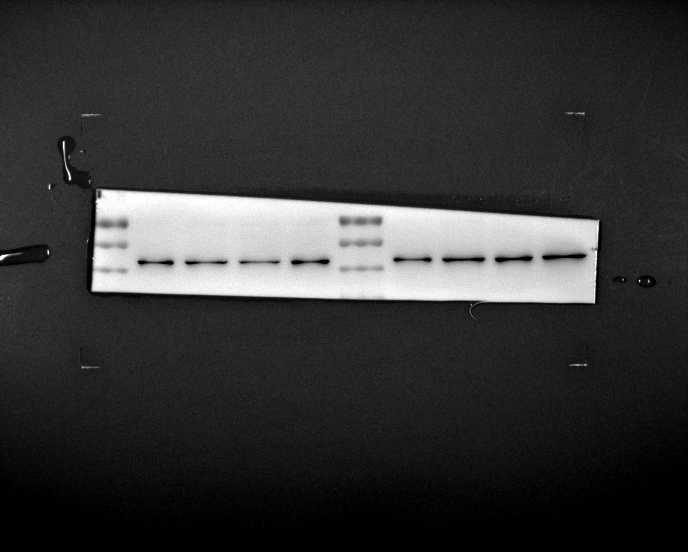

Supplement: Supplementary file 1 [file Data_Sheet_1.ZIP › Fig.4D TX-100-beta-actin.tif]

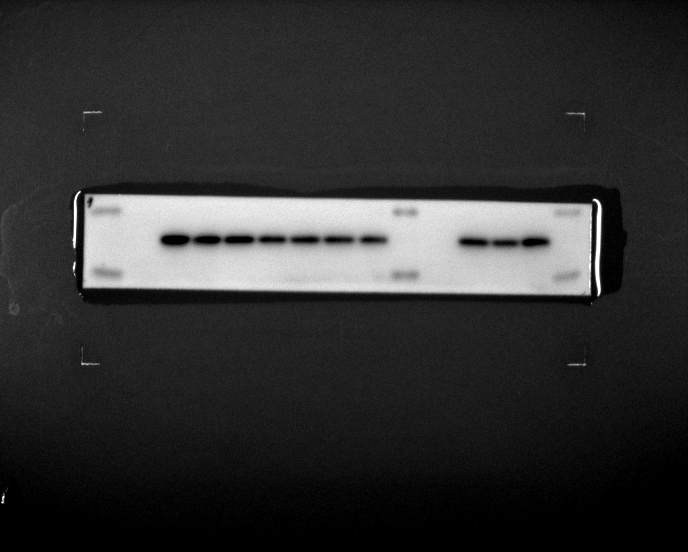

Supplement: Supplementary file 1 [file Data_Sheet_1.ZIP › Fig.4G aSyn.tif]

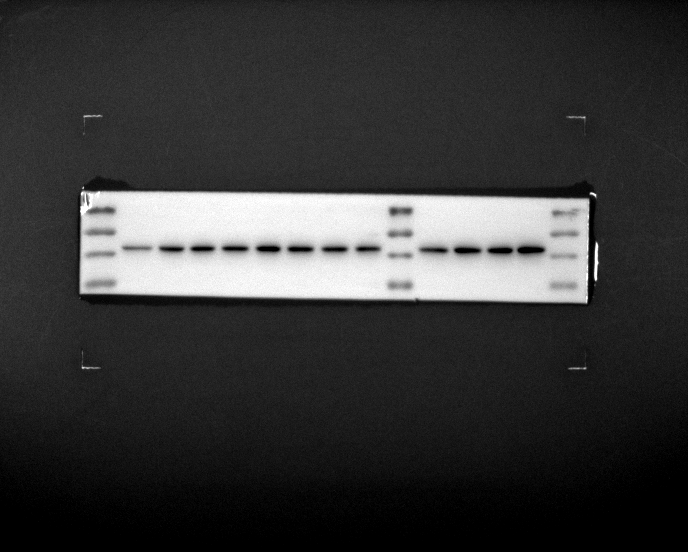

Supplement: Supplementary file 1 [file Data_Sheet_1.ZIP › Fig.4G beta-actin.tif]

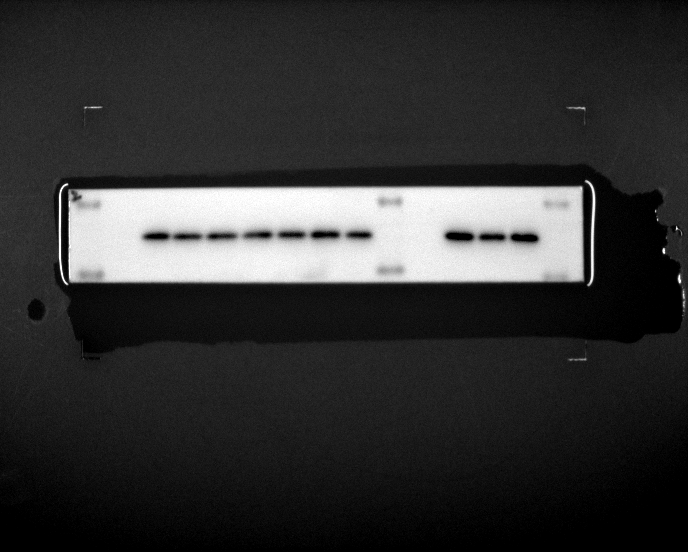

Supplement: Supplementary file 1 [file Data_Sheet_1.ZIP › Fig.4G GFP.tif]

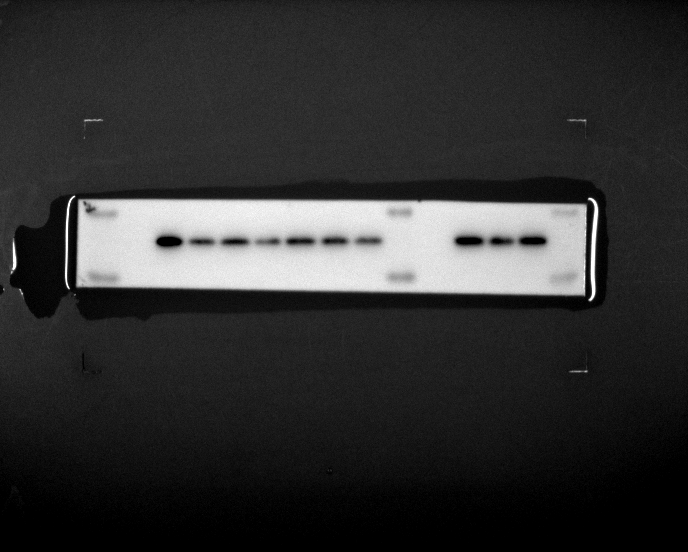

Supplement: Supplementary file 1 [file Data_Sheet_1.ZIP › Fig.4G His-tag.tif]

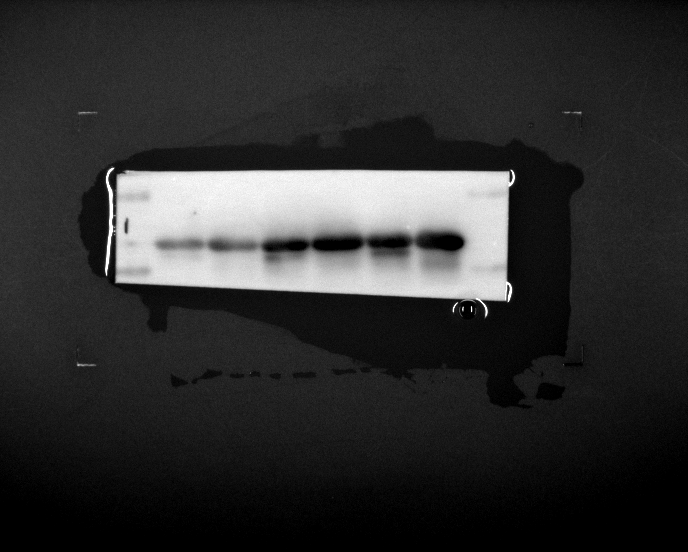

Supplement: Supplementary file 1 [file Data_Sheet_1.ZIP › spFig.1A haSyn-merge.tif]

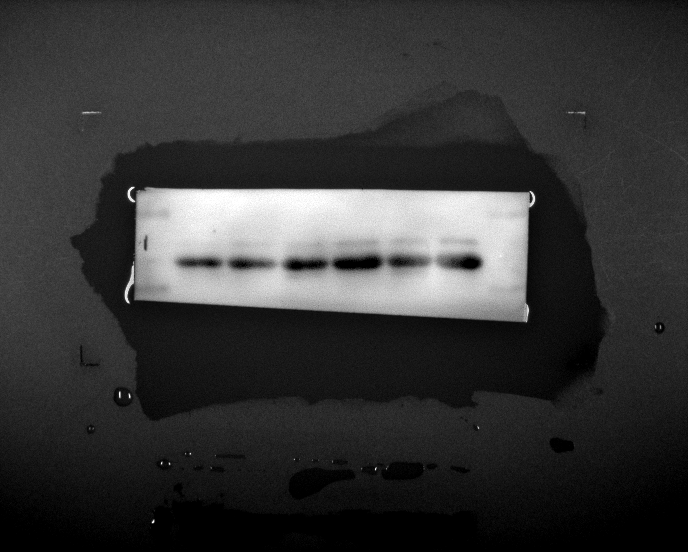

Supplement: Supplementary file 1 [file Data_Sheet_1.ZIP › spFig.1A His-tag-merge.tif]

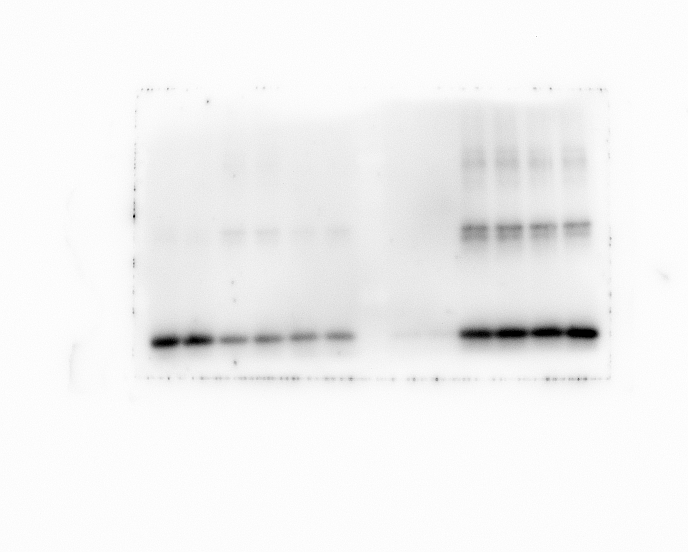

Supplement: Supplementary file 1 [file Data_Sheet_1.ZIP › spFig.1D.tif]

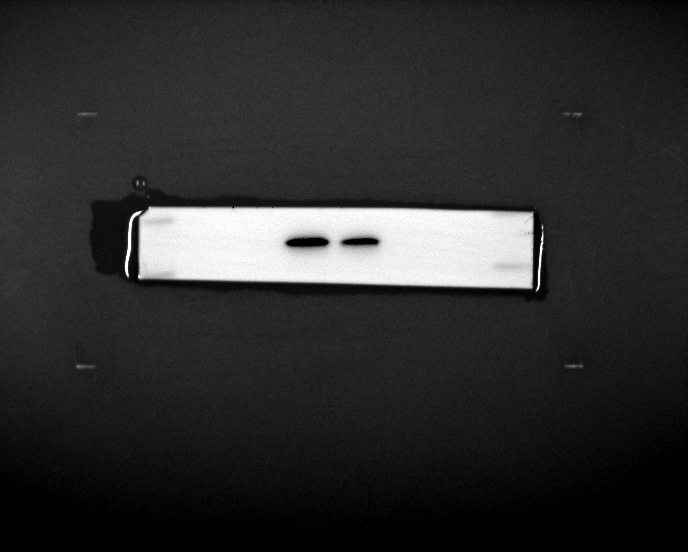

Supplement: Supplementary file 1 [file Data_Sheet_1.ZIP › spFig.2A aSyn.tif]

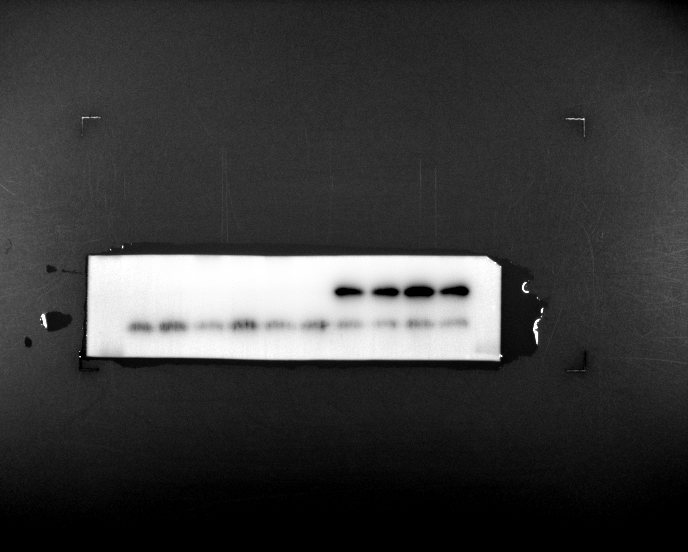

Supplement: Supplementary file 1 [file Data_Sheet_1.ZIP › spFig.2A GFP.tif]

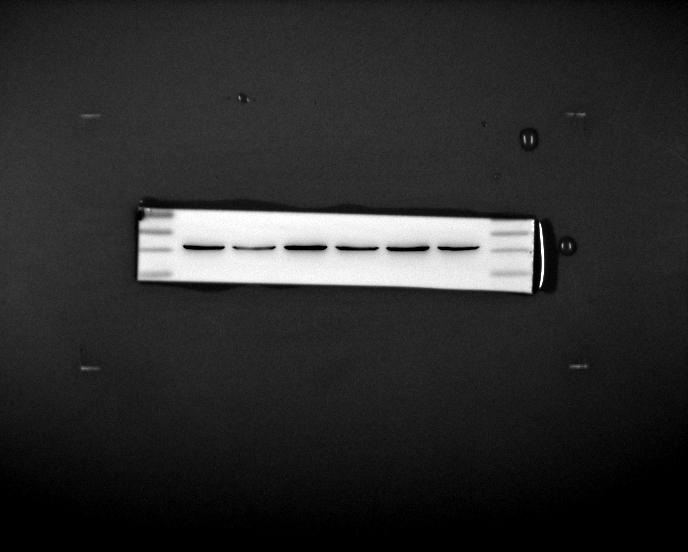

Supplement: Supplementary file 1 [file Data_Sheet_1.ZIP › spFig.2A β-actin.tif]

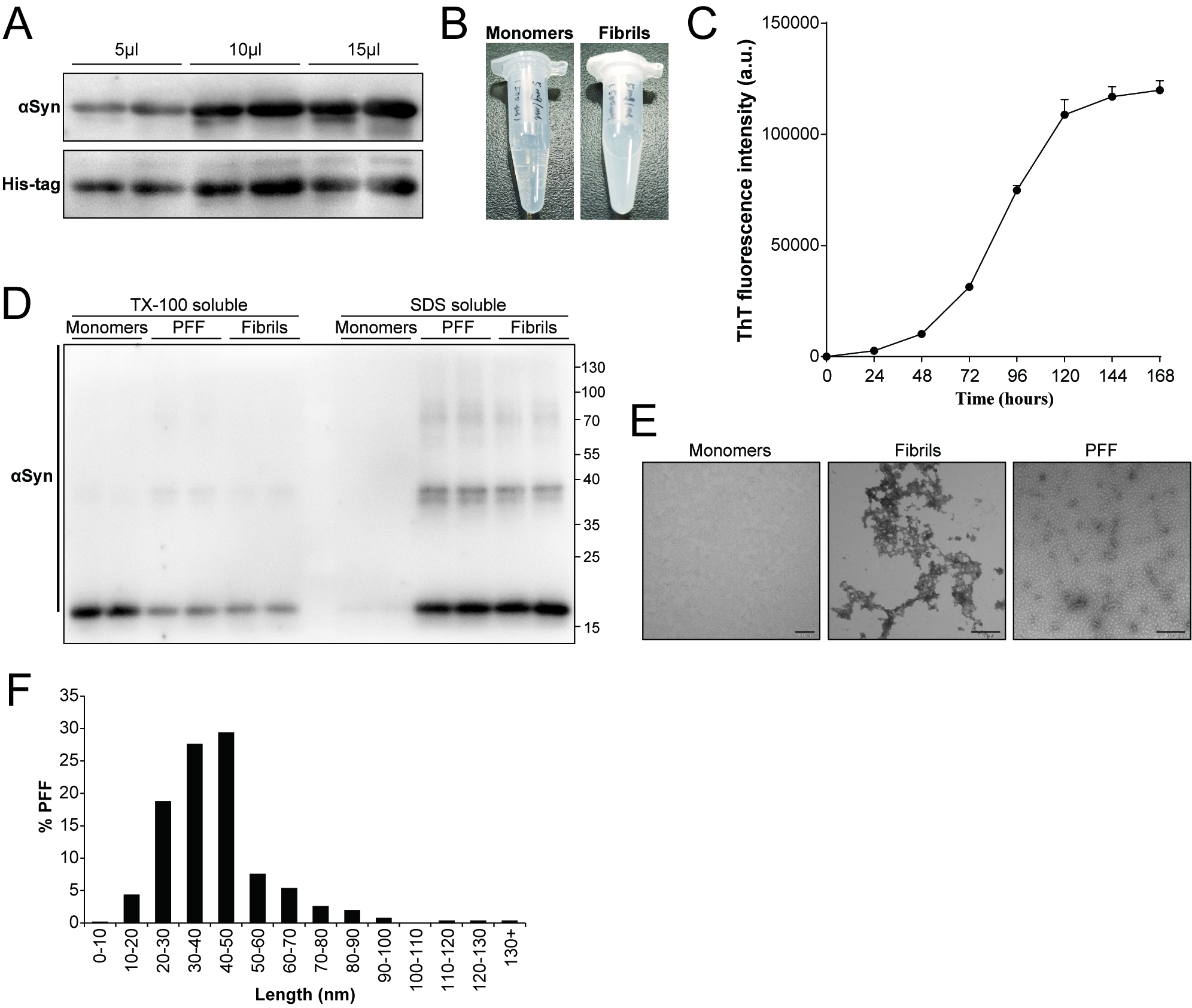

Supplement: Supplementary file 2 [file Image_1.TIF]

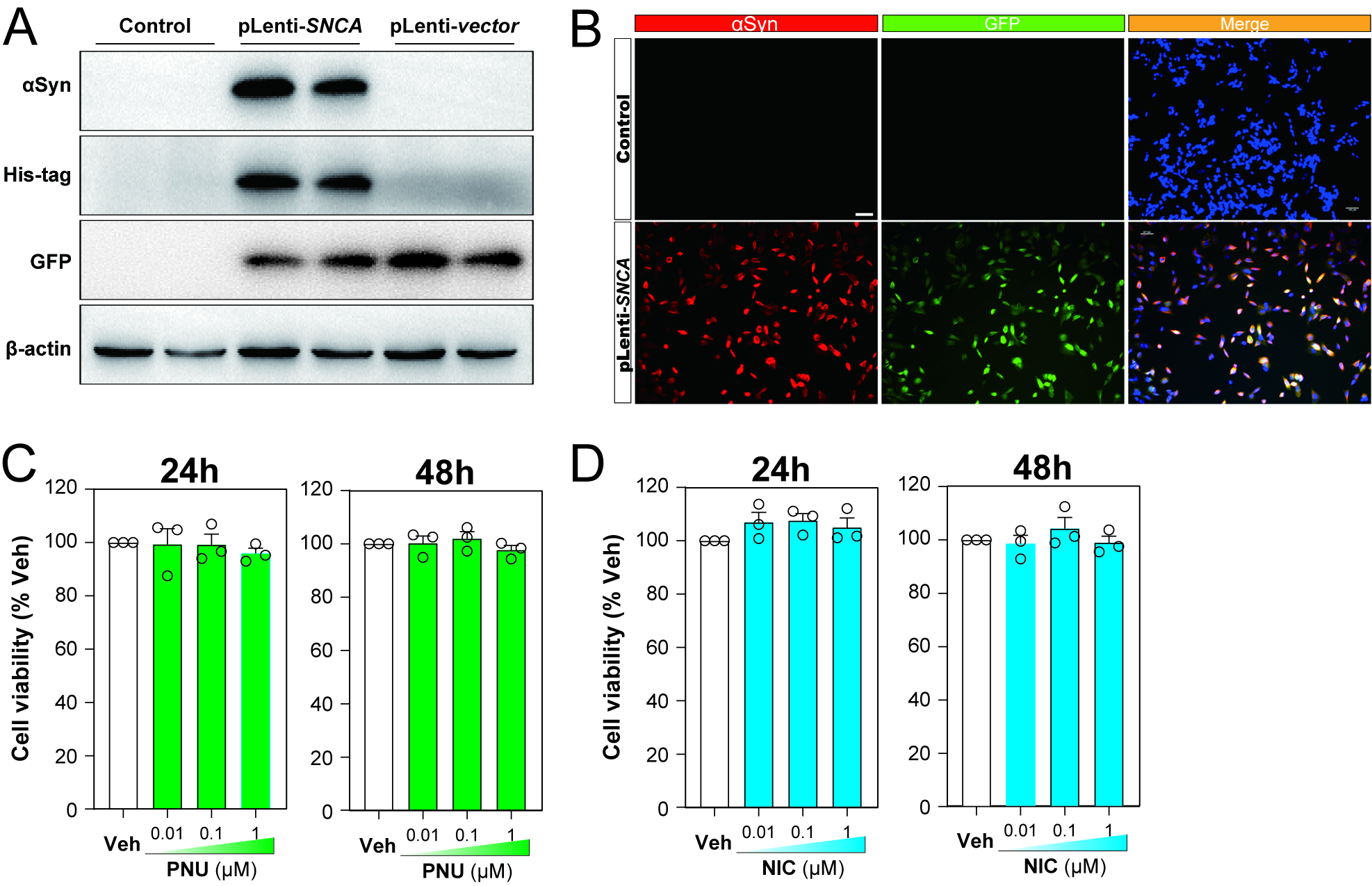

Supplement: Supplementary file 3 [file Image_2.TIF]
